# Reliability, feasibility, and validity of the quality of interactions schedule (QuIS) in acute hospital care: an observational study

**DOI:** 10.1186/s12913-017-2312-2

**Published:** 2017-05-31

**Authors:** Christopher McLean, Peter Griffiths, Ines Mesa – Eguiagaray, Ruth M. Pickering, Jackie Bridges

**Affiliations:** 1 Faculty of Health Sciences, University of Southampton, Southampton, UK; 2 Faculty of Medicine, University of Southampton, Southampton, UK

**Keywords:** Acute care, Communication, Interpersonal relations, Quality of interactions schedule, QuIS, Outcome measures, Validation studies

## Abstract

**Background:**

Research into relational care in hospitals will be facilitated by a focus on staff-patient interactions. The Quality of Interactions Schedule (QuIS) uses independent observers to measure the number of staff-patient interactions within a healthcare context, and to rate these interactions as ‘positive social’; ‘positive care’; ‘neutral’; ‘negative protective’; or ‘negative restrictive’. QuIS was developed as a research instrument in long term care settings and has since been used for quality improvement in acute care. Prior to this study, its use had not been standardised, and reliability and validity in acute care had not been established.

**Methods:**

In 2014 and 2015 a three - phase study was undertaken to develop and test protocols for the use of QuIS across three acute wards within one NHS trust in England. The phases were: (1) A pilot of 16 h observation which developed implementation strategies for QuIS in this context; (2) training two observers and undertaking 16 h of paired observation to inform the development of training protocols; (3) training four nurses and two lay volunteers according to a finalised protocol followed by 36 h of paired observations to test inter-rater agreement. Additionally, patients were asked to rate interactions and to complete a shortened version of the Patient Evaluation of Emotional Care during Hospitalisation (PEECH) questionnaire.

**Results:**

Protocols were developed for the use of QuIS in acute care. Patients experienced an average of 6.7 interactions/patient/h (*n* = 447 interactions). There was close agreement between observers in relation to the number of interactions observed (Intraclass correlation coefficient (ICC) = 0.97) and moderate to substantial agreement on the quality of interactions (absolute agreement 73%, kappa 0.53 to 0.62 depending on weighting scheme). There was 79% agreement (weighted kappa 0.40: *P* < 0.001; indicating fair agreement) between patients and observers over whether interactions were positive, negative or neutral.

**Conclusions:**

Observers using clear QuIS protocols can achieve levels of agreement that are acceptable for the use of QuIS as a research instrument. There is fair agreement between observers and patients’ rating of interactions. Further research is needed to explore the relationship between QuIS measures and reported patient experience.

**Electronic supplementary material:**

The online version of this article (doi:10.1186/s12913-017-2312-2) contains supplementary material, which is available to authorized users.

## Background

There is widespread international concern that staff-patient interactions within acute hospitals do not always promote compassionate and high quality relational care [1–3]. Whilst interventions can be designed and implemented which aim to promote such care [4, 5], measuring the impact of these interventions is challenging and the development of appropriate measures has been limited to date. Relational care and compassion are demonstrated through interactions between healthcare staff and service users [4] and so the quality of staff-patient interactions is an important and appropriate focus for healthcare research. The Quality of Interactions Schedule (QuIS) was developed by Dean et al in 1993 [6] to measure the quality and quantity of staff-patient interactions in long term care settings. Although QuIS is a reliable instrument within this context [6], its use has not been standardised and its reliability and validity in acute care has not been established. This paper reports a study which examined the feasibility of developing QuIS for use in acute care settings.

Whilst some other research instruments or service development tools begin to explore the quality of staff-patient interactions, all are problematic as definitive measures. Common measures of patient experience such as the ‘Patient Evaluation of Emotional Care during Hospitalisation’ (PEECH) [7] or Picker Patient Experience (PPE-15) questionnaires [8] focus on patients’ overall experience of hospitalisation rather than on individual interactions and rely on the ability of patients to self-report. Those tools which do focus on the quality of individual staff-patient interactions such as dementia care mapping (DCM) [9], or the Person Interaction Environment (PIE) approach used within the National Audit of Dementia [10] have been developed for educational and service improvement purposes and their use as a research instrument is problematic. The PIE tool lacks a structure suitable for quantitative use, whilst DCM has weak psychometric properties and is resource-intensive to use [11].

Studies which attempted to quantify the frequency, duration and classified type of nurse patient interaction were prevalent in the UK through the 1960’s to 1990’s [12], though more recent literature is lacking. The Quality of Interactions Schedule has origins in observational research undertaken in 1989 by Clark & Bowling [13] in which interactions were rated as either ‘positive’, ‘negative’ or ‘neutral’. In 1993, Dean et al used this work to develop the Quality of Interaction Schedule which introduced distinctions within the ‘positive’ and’negative’ categories and proposed a five category scale set out in Table 1 [6]. QuIS is now generally regarded as an ordinal scale ranging from the highest ranking Positive Social interactions to the lowest ranking Negative Restrictive interactions [14].

**Table 1:** Definitions of QuIS categories from Dean et al 1993

Positive social	Interaction principally involving ‘good, constructive, beneficial’ conversation and companionship
Positive Care	Interactions during the appropriate delivery of physical care
Neutral	Brief, indifferent interactions not meeting the definitions of the other categories
Negative protective	Providing care, keeping safe or removing from danger, but in a restrictive manner, without explanation or reassurance: in a way which disregards dignity or fails to demonstrate respect for the individual
Negative restrictive	Interactions that oppose or resist peoples’ freedom of action without good reason, or which ignore them as a person

Searches of CINAHL, EMBASE and MEDLINE databases revealed 8 studies that have used QuIS as an outcome measure [15–22] although none were conducted on patients with somatic illness in acute hospitals. The use of QuIS as a research tool has been restricted to community and long term care settings, although QuIS has been used as a tool to promote service improvement (rather than as an outcome measure) within acute care settings [23–25].

Research studies which report using QuIS [15–22] demonstrate considerable variation in the way in which the five categories of interaction have been defined and exemplified. Further variation is apparent within service improvement projects where teams have often used significantly different categories from those proposed by Dean et al (1993) [6]. For example, the ‘Leadership in Compassionate care programme’ [24] used ‘QuIS’ with only three categories of interaction (positive, neutral, negative), whilst the 2010 Dignity in Care project [25] used a shortened QuIS (S-QuIS) with four categories (positive social, positive care; neutral and negative). This wide variation highlights the need to develop and test the reliability of standardised descriptors and exemplars for QuIS categories.

Review of prior studies also demonstrates variation in other elements of QuIS protocols such as time-sampling approaches which range from sampling periods of 30 s [15] to up to three hours [18]. Whilst most researchers have focussed on staff- patient interactions, some research teams have also recorded patient—patient interactions [15]. Similar variation exists in relation to whether observation focussed on the interactions experienced by one or more service users, the interactions engaged in by one member of staff; or on all interactions within a geographical area. These variations further highlight a need for standardised protocols in order for QuIS data to be comparable across studies.

Inter-rater reliability studies for QuIS have generally demonstrated high levels of agreement by reporting Cohen’s kappa. It has been proposed that kappa agreement may be described as ‘poor’ (kappa ≤ 0); ‘slight’ (kappa 0.01–0.20); ‘fair’ (kappa 0.21–0.40); ‘moderate’ (kappa 0.41–0.60); ‘substantial’ (kappa 0.61–0.80); or ‘almost perfect’ (kappa 0.81–1.0) [26]. In the original Dean et al study [6] kappa was reported as > 0.7 (‘substantial’ agreement), whilst in 1998 Jenkins and Allen [15] reported kappa of 0.80 to 0.96 (‘almost perfect’ agreement). However, in these studies reliability was largely tested by asking a second rater to categorise interactions based upon written descriptions produced by the first observer. Dean et al [6] is the only study to have also tested inter-rater reliability by having two observers rate 148 interactions as they actually happened, and found that agreement to be more variable, although still acceptable (kappa 0.60 – 0.91).

QuIS has been reported to be sensitive to changes in service quality [6, 27]. Studies using QuIS have argued or assumed that there is a relationship between an increased number of positive interactions (or decreased number of negative interactions) and positive patient experience although none have directly demonstrated this. There is a need to understand whether there is a relationship between patient experience and the number and/or quality of interactions measured by QuIS.

This study aimed to determine whether it is feasible to use QuIS to measure the quality of interactions in an acute care setting. In order to achieve this aim we set objectives to:

1. Identify practical obstacles to the use of QuIS in acute care and incorporate solutions within detailed observational protocols.
2. Develop and test training protocols for the use of QuIS measures in acute care.
3. Determine inter-rater reliability for the number and quality of interactions recorded using these protocols.
4. Explore the relationship between patient reported experience and observer categorisations of interactions.

## Methods

### Overview of study design

The study took a three stage iterative approach to the testing, revision, and retesting of observational and training protocols for the use of QuIS in acute care. The study was undertaken in three medical wards within one Acute NHS trust in England (An Acute Medical Unit (AMU), one ward specialising in Medicine for Older People, and one Hepatology ward).

In the first stage, 14 h of observation were undertaken by a single investigator (Observer 1) in order to identify practical challenges and potential solutions to the use of QuIS in acute care. In the second stage, two additional observers (Observers 2 & 3) were trained according to preliminary protocols. Each of these observers undertook four two-hour periods of observation paired with Observer 1 (hence 16 h of observation in total). This stage enabled observers to identify and discuss discrepancies in the number and rating of observed interactions so as to develop and refine protocols and training.

In the third stage, an additional four observers (Observers 4–7) were recruited and trained according to a finalised protocol. In this phase a further 18 periods of paired observation were undertaken according to a schedule which maximised variation in the pairing of Observers 1–7. In order to capture the patient perspective on interactions, patients provided their own evaluation of specific interactions, and completed a shortened version of the Patient Evaluation of Emotional Care during Hospitalisation (PEECH) questionnaire [7].

### Sampling and recruitment

In addition to the primary investigator (CM), four nurses and two non-clinicians were recruited as observers. The nurses had a minimum of 18 months post registration experience, and comprised two staff nurses, one clinical educator and one matron. None of the nurses worked in the wards in which data were being collected. The two non-clinical observers were hospital volunteers already registered with the Trust who offered to participate in response to information circulated in a volunteer newsletter.

Observation was undertaken between the hours of 08.00 and 18.00 during Monday to Friday. Patients were purposefully selected in order to achieve maximum variation in the contexts in which observation was undertaken (such as the geographical features of individual wards/bays), and to maximise the number of interactions observed (e.g. selecting patients who were not anticipated to leave the ward for medical investigations/discharge). Patients without capacity to consent were excluded, as were patients where staff identified clinical reasons why inclusion might have been inappropriate (such as acute deterioration in their condition necessitating continual presence of staff).

### Data collection

#### QuIS protocol

Observer training addressed key challenges of undertaking observational research, particularly the need for observers to anticipate, identify and respond appropriately to unexpected events during observation such as patient anxiety, dangerous practice, or patient safety breaches. Observers adopted a purely observational role and wore plain clothes to collect data. The core definitions of each QuIS category (Table 1) were taken from the original 1993 study by Dean et al [6]. Detailed guidance and exemplars were developed which guided observers in relation to (a) defining interactions, and (b) applying these definitions to the range of interactions encountered in acute care.

An interaction was considered to be any action or speech between staff and patients of which the patient was aware. Hence patient to patient communications were not considered, whilst communications between staff members were considered interactions if occurring in the immediate vicinity of the patient. An interaction was considered to begin (or end) when staff or patients demonstrably directed attention towards (or away from) one another through speech or non-verbal communication; or by staff entering/leaving the immediate proximity of the patient delineated by the curtains around the bed (for multi bedded rooms).

**Fig. 1:**
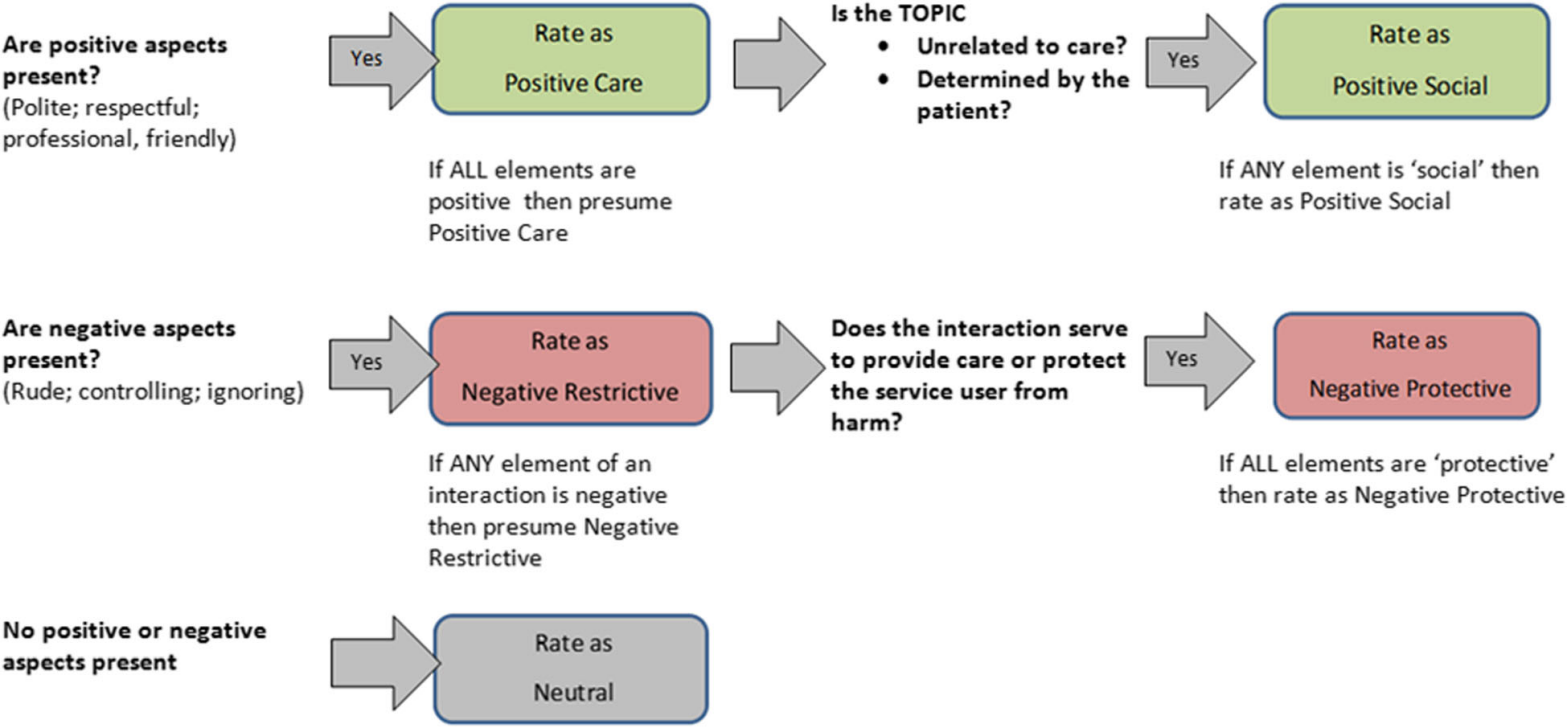
Flow chart summary of QuIS protocol

The original definitions of QuIS categories [6] do not provide examples that are relevant to the full range of staff-patient interactions encountered in acute care, so guidance was developed on how these definitions could be applied within acute care. During the first two stages, observers found it difficult to consistently differentiate Positive Care and Positive Social interactions, as they could disagree over whether staff gave explanation or encouragement that was “more than necessary to carry out the task” (which is a characteristic of Positive Social interactions according to Dean et al [6]). Our protocol distinguished between Positive Care and Positive Social interactions primarily on the basis of the topic of conversation. Whilst many interactions relate solely to patients acute care needs (Positive Care interactions), discussion of non-care related topics such as family, holidays, or other outside interests was considered to represent the “conversation and companionship” which characterise Positive Social interactions [6]. This distinction reflected an interpretation that Positive Social interactions may reinforce the service users’ identity as a ‘*person*’, whilst Positive Care interactions may reinforce their identity as a ‘*patient*’. These principles were incorporated into a flow chart summary which served as an aide memoire to these elements of the protocol (see Fig. 1).

Training also emphasised principles such as the need to focus on the patient perspective and to avoid making allowances for staff. Training also offered guidance in relation to commonly encountered special cases such as brief greetings or offers of hotel services such as food and drink. The protocol also incorporated a means of managing uncertainty given that the default rating for positively or negative perceived interactions was Positive Care or Negative Restrictive respectively (see Fig. 1).

#### Additional data

In addition to QuIS category, the data collected included the start and finish time of each interaction (to the nearest minute); staff groups and number of staff involved; and a summary of the content of the interaction. During the first two phases of the study, additional freehand notes were maintained in order to record challenges or obstacles to the use of QuIS which informed the development of observational and training protocols and provided vignettes which were anonymised for incorporation into training materials.

Immediately after each period of observation a unique identification number for each interaction was generated through discussion between the observers. Interactions were taken to be identical (and hence given the same interaction number) where the patient involved, start time, and content of the interaction were the same for each observer. This approach ensured that analysis could distinguish between agreement over the number of unique interactions recorded, and agreement over the QuIS rating of an identified interaction.

Whilst results are reported for ‘Observer 1’ and ‘Observer 2’ these headings do not relate to individuals, but rather serve to highlight the overall agreement between observers that was achieved. The most experienced observer for each session was considered ‘Observer 1’ and less experienced observer was considered ‘Observer 2’.

Within one hour of the observation ending, patients who had capacity to recall specific interactions were asked to give their own rating of the observed interactions. Patients were asked firstly whether they could remember each interaction, and if so to rate these as ‘positive’, ‘neutral’ or ‘negative’. Patients also completed the ‘personal value’ subscale of the Patient Evaluation of Emotional Care during Hospitalisation (PEECH) questionnaire [7] based upon how they experienced interactions during the period of observation. Questions within this subscale of the PEECH instrument are known to have a particularly strong correlation with summary measures of patient experience [28] and have particular relevance to the experience of individual interactions.

#### Analysis

Data were analysed using Stata 11 [29] and the threshold for statistical significance was set at 0.05. The length of interactions was calculated to the nearest minute by examining the difference between the recorded start and finish times of interactions, and when raters disagreed we used the average length of interaction. Agreement in the number and length of interactions was described using absolute difference and intra-class correlation coefficients with 95% confidence intervals. Where pairs of observed interactions could be matched, agreement between the two observers on the QuIS category was described in terms of absolute (%) agreement and the kappa coefficient (indicating agreement above chance). We calculated unweighted and weighted kappas (reflecting the degree of disagreement based on the similarity between QuIS categories), and linear weighted kappa between QuIS (collapsed to 3 points) and patient evaluations. Unweighted and weighted kappa estimates were calculated for each of the 18 observation periods and combined into a single overall summary kappa using a random effects approach [30]. In order to explore the relationship between patient reported experience and observer QuIS ratings of interactions, Pearson’s correlation coefficients were calculated between mean patient scores for the personal value subscale of the PEECH questionnaire and (1) the percentage of interactions rated QuIS positive averaged across the two observes, and (2) the total number of interactions experienced by the patient. Spearman’s correlation coefficients were calculated in relation to the individual PEECH items and the percentages of interactions rated QuIS positive.

#### Ethics

Written consent was obtained from patients prior to conducting observation, and the presence of observers was also explained to non-participating patients and visitors in the vicinity. All patient information was anonymised within the data. Staff were made aware of the study through discussion at team meetings, and through the provision of posters and information sheets sent via email as well as being available in hard copy. Staff present at the time of observations were given opportunities to ask questions and/or to decline to participate.

## Results

Eighteen periods of observation were completed in the final phase of the study, each of which lasted for two hours and involved the observation of two patients. In each session, two observers recorded details of all interactions between staff and the identified patients. Four hundred and forty-seven interactions were observed, of which 354 (79%) were witnessed by both observers (see Additional file 1). Although observers did not always record the same interactions, the total number of interactions recorded by observers was nevertheless very similar (Intraclass correlation coefficient (ICC) = 0.97: 95% Confidence Interval: 0.92 – 0.99, *P* <0.001). The occasional absence of patients from ward areas for short periods means that interactions were recorded for 67 patient hours, resulting in a mean interaction frequency of 6.7 interactions/patient/h.

The mean recorded duration of interactions was 1.6 min (median = 0.5), and 259 (58%) interactions were of one minute or less in duration. There was perfect agreement on interaction length for 224 of the 354 (63%) jointly observed interactions. Overall agreement for the length of interaction recorded by different observers was moderate with a mean absolute difference of 0.4 min (ICC = 0.56; 95% CI 0.48 – 0.62: *P* <0.001). Duration of interactions varied according to the staff group involved. Doctors were involved in the longest interactions (mean duration 4.5 min) whilst the shortest interactions (mean duration 0.9 min) involved “other staff” which included allied health professionals, domestic and ancillary staff. Mean duration of nurse-patient interactions was 1.8 min.

Observers attributed the same QuIS category to 260 (73%) of the 354 interactions witnessed by two observers. The unweighted kappa was 0.53 (moderate agreement). Agreement is shown in detail in Table 2.

**Table 2:** Observer agreement for QuIS categories (combined over 18 observation periods)

	Observer 1						
		Positive social	Positive care	Neutral	Negative protective	Negative restrictive	Total
Observer 2	Positive social	36	22	3	0	3	64
	Positive care	23	164	13	5	1	206
	Neutral	0	10	47	2	0	59
	Negative protective	0	4	2	7	0	13
	Negative restrictive	0	1	5	0	6	12
	TOTAL	59	201	70	14	10	354

Weighted kappa were calculated using a number of different weightings. Giving a weight of 0.75 to agreements within positive and negative categories, 0.5 where one rater gave a neutral rating and 0 for any disagreement on positive/negative categories yielded a weighted kappa of 0.57 (moderate agreement). All weighted Kappas lay in the range 0.56– 0.62 (moderate to substantial agreement), with the highest weighted kappa of 0.62 achieved by attributing equal weighting to the two positive and two negative categories (equivalent to testing for agreement on a collapsed 3-point scale) (see Table 3).

**Table 3:** Inter-rater agreement measured by kappa using various weighting schemes (combined over 18 observation periods)

Method		Weights					KAPPA 95% CI
		+ s	+ c	N	- p	- r	
Unweighted	+ social	1					0.53 (0.45, 0.60)
	+ care	0	1				
	Neutral	0	0	1			
	- protective	0	0	0	1		
	- restrictive	0	0	0	0	1	
Equal weighting given ignoring differences within + ve categories, and within –ve categories (equivalent to testing agreement on a 3-point scale)	+ social	1					0.62 (0.48, 0.77)
	+ care	1	1				
	Neutral	0.5	0.5	1			
	- protective	0	0	0.5	1		
	- restrictive	0	0	0.5	1	1	
Weighted (linear weights reflecting ordinality with equal spacing)	+ social	1					0.56 (0.46, 0.66)
	+ care	0.75	1				
	Neutral	0.5	0.75	1			
	- protective	0.25	0.5	0.75	1		
	- restrictive	0	0.25	0.5	0.75	1	
Weightings given to neutral compared to a positive or negative = 0.5, assuming that disagreement between the positives is equal to disagreement between the negatives							
Weighted 1	+ social	1					0.60 (0.47, 0.73)
	+ care	0.9	1				
	Neutral	0.5	0.5	1			
	- protective	0	0	0.5	1		
	- restrictive	0	0	0.5	0.9	1	
Weighted 2	+ social	1					0.57 (0.47, 0.68)
	+ care	0.75	1				
	Neutral	0.5	0.5	1			
	- protective	0	0	0.5	1		
	- restrictive	0	0	0.5	0.75	1	
Weighted 3	+ social	1					0.55 (0.46, 0.64)
	+ care	0.6	1				
	Neutral	0.5	0.5	1			
	- protective	0	0	0.5	1		
	- restrictive	0	0	0.5	0.6	1	

Patient perspectives on interactions were sought from 17 patients for 185 (41%) of the interactions observed, 30 (16%) of which could not be recalled by the patient involved. Of the 155 interactions rated by patients, 130 (84%) were rated as positive compared with 120 (77%) which were rated positively by independent observers. Patients rated 19 (12%) interactions as neutral and six (4%) as negative. There was 79% agreement (kappa 0.40: P < 0.001; indicating fair agreement) between patients and observers over whether interactions were positive, negative or neutral. (Table 4).

**Table 4:** Agreement between patient and observer rating of interactions

		QUIS rating for observer 1 (collapsed to 3 category scale)			
		Positive	Neutral	Negative	Total
Patient evaluation	positive	112	16	2	130
	neutral	6	9	4	19
	negative	2	3	1	6
Total		120	28	7	155

Eighteen patients (58%) were able to complete the shortened PEECH questionnaire. Sixteen of these patients completed all questions, whilst two patients answered only nine of 10 available questions. The mean PEECH score for Personal Value was 2.47 out of a possible maximum of 4 (SD 0.51). No significant correlations were noted between overall PEECH scores and the total number of interactions experienced by patients. A moderate correlation between the percentage of interactions rated positively by observers and the mean PEECH score did not reach statistical significance (Pearson’s correlation *r* = 0.42; *P* =0.086).

There was significant correlation between the percentage of interactions which were rated positively and one individual PEECH item “exceeded expectations” (Spearman’s *r* = 0.603, *P* = 0.008). There was moderate association between the percentage of positively rated interactions and two other individual PEECH items, these being “facial expression” (Spearman’s *r* = 0.426, *P* = 0.088) and “social conversation” (Spearman’s *r* = 0.402, *P* = 0.098), neither of which achieved statistical significance (Table 5).

**Table 5:** Spearman’s correlation coefficients between individual PEECH questions and percentage of positive QuIS ratings

PEECH items	Averaged % of QuIS categorisations which were positive (Combined for both observers)	
	Coefficient	*P* value
The staff used appropriate eye contact when communicating with me	0.006	0.982
The staff were neither too close nor too far away when they communicated with me	-0.260	0.298
The staff used an appropriate tone of voice when they communicated with me	0.218	0.385
The staff displayed gentleness and concern when they cared for me	0.012	0.962
The staff encouraged me when I needed support	0.168	0.520
I felt that the staff really listened to me when I talked	-0.111	0.660
The care that I have received from the staff has exceeded my expectations	**0.603**	**0.008**
The staff used appropriate facial expressions when communicating with me	0.426	0.088
The staff engaged me in social topics of conversation at suitable times	0.402	0.098
I felt valued as a person during this admission	-0.114	0.653

Bold indicates significance where p is taken as < 0.05

No formal attempt was made to quantify or characterise observer effects, though staff were asked to comment on whether they felt the experience of being observed had influenced their practice. The majority of staff stated that their practice had not been influenced by being observed, though some reported that observation made them feel awkward or self-conscious such that their interactions were more constrained and less ‘chatty’ than normal.

## Discussion

Within the initial two phases of this study we found QuIS to be feasible for use in acute settings, and developed observational and training protocols for such use. The final stage of the study aimed to determine inter-rater reliability for the number and quality of interactions recorded using these protocols, and explore the relationship between patient reported experience and observer ratings of interactions. Our results indicate that patients experienced an average of 6.7 interactions per hour with staff and that observers achieve close agreement on the total number of interactions occurring, though there was less agreement on the length of these interactions. Observers agreed on QuIS category in 73% of interactions with kappa co-efficient of 0.53 – 0.62, indicating moderate to substantial agreement depending on the weighting used. There was fair agreement between patients’ rating of interactions they recalled and observer ratings. There was some evidence of correlation between the proportion of positive interactions and patients’ positive rating of experiences.

Although other studies have reported higher agreement than reported here, only one prior study has tested the reliability of QuIS by asking independent observers to rate interactions in situ [6]. Our results give comparable although slightly lower estimates of inter-rater reliability. Unweighted kappa treats all disagreement as equal and does not take into account the degree of disagreement between observers. Whilst QuIS may be considered a ranked ordinal scale [14], this is not a straightforward assumption given that the distinction between ‘positive’, ‘neutral’ and ‘negative’ interactions may be considered of greater importance than the relatively minor distinctions within these categories. In our study, values for weighted kappa were calculated using a number of different weightings which reflected a range of assumptions, though it is notable that variation in the weightings attributed had only a modest impact on the resultant estimate of agreement. A balanced choice (with disagreements between neutral and either positive or negative weighted 0.5 and disagreements within positive or negative weighted as 0.75) gives a kappa of 0.57 (95% CI: 0.47 – 0.68), which is considered moderate agreement.

Overall we found only low levels of agreement between observers on the length of interactions. These findings may reflect the cognitive load placed on observers who occasionally reported that they had ‘missed’ the end of interactions. Further development of QuIS would be aided by the development of methods of data collection which maximise the accuracy with which the length of interaction may be recorded. A further factor which influences agreement on the length of interaction is the criteria used to differentiate interactions as unique, and this may be made clear by considering an example. A healthcare worker who spends five minutes with a patient but who is briefly interrupted or responds to other patients on two occasions may be recorded to have taken part in one five minute long interaction, or in three much briefer interactions. This example highlights the need for consistent criteria, and makes clear that there is a relationship between the number of interactions recorded and the length of those interactions. These considerations also highlight the complexity of interpreting such data and suggest that total time spent in interactions (i.e. length of interaction multiplied by the number of interactions) could be important when reporting QuIS data.

In order to explore the relationship between patient reported experience and observer categorisations of interactions patients were asked to give their own evaluation of interactions and to complete the shortened PEECH questionnaire. It was possible to seek patients’ own evaluation of interactions for only 41% of interactions, and to obtain PEECH data from only 18 patients. This low response rate may be partially attributed to the fact that a greater percentage of patients than was anticipated were experiencing some degree of cognitive impairment such that, whilst able to consent and participate in the study, they could not retrospectively recall specific interactions. While a limitation this illustrates a potential advantage of QuIS in that the experience of these patients can potentially be considered whereas other methods would exclude them. We found fair agreement (weighted kappa = 0.40) between patients and observers over whether interactions were positive, negative or neutral.

No statistically significant relationship was found between overall scores on the shortened PEECH questionnaire and the percentage of interactions which were rated as QuIS positive by independent observers. There was significant association between the percentage of positive interactions and positive evaluations on only one individual PEECH item and since the analysis should be considered as exploratory this might be a chance finding. No correlation was found between QuIS ratings and PEECH items which may be particularly considered indicative of compassion such as whether staff displayed ‘gentleness and concern’. PEECH is an overall measure of patient experience and may measure a different construct than QuIS which focuses on discreet individual interactions. These findings are also limited by the sample size available. Further research is needed in order to explore the relationship between the quality of staff-patient interactions and patients’ experience of acute hospital care.

The presence of observers may itself impact on the frequency or quality of staff patient interactions. Although no formal attempt was made to quantify or characterise these observer effects, our experiences in this study suggest that they should not be presumed to artificially *increase* the quality of interactions. Given that some staff reported their interactions to be more constrained and less ‘chatty’ than normal, it is possible that QuIS may underestimate the percentage of ‘Positive Social’ interactions.

## Conclusions

Using the Quality of Interactions Schedule (QuIS) to categorise staff-patient interactions within acute hospital settings is feasible and workable. The protocols developed to guide the use of QuIS within this setting enable reliable observation by trained observers. There is some evidence that QuIS ratings correlate with patient ratings of quality and their reports of experience. These results support the use of QuIS as a measure of the quality of staff-patient interactions in acute care settings, particularly given the absence of alternative definitive tools. Further research is needed in order to explore the relationship between QuIS measures and reported patient experience.

## Additional file

Additional file 1: Data file. (XLSX 54 kb).

## Abbreviations

PEECH: Patient evaluation of emotional care during hospitalisation
QuIS: Quality of interactions schedule
